# Study of In-Vehicle Ethernet Message Scheduling Based on the Adaptive Frame Segmentation Algorithm

**DOI:** 10.3390/s25082522

**Published:** 2025-04-17

**Authors:** Jiaoyue Chen, Yujing Wu, Yihu Xu, Kaihang Zhang, Yinan Xu

**Affiliations:** College of Engineering, Yanbian University, Yanji 133002, China; 2023050038@ybu.edu.cn (J.C.); yjwu@ybu.edu.cn (Y.W.); xuyh@ybu.edu.cn (Y.X.); 2689332993@ybu.edu.cn (K.Z.)

**Keywords:** bandwidth utilization, in-vehicle ethernet, adaptive frame segmentation, message scheduling, time-sensitive networking

## Abstract

With the rapid development of intelligent driving technology, in-vehicle bus networks face increasingly stringent requirements for real-time performance and data transmission. Traditional bus network technologies such as LIN, CAN, and FlexRay are showing significant limitations in terms of bandwidth and response speed. In-Vehicle Ethernet, with its advantages of high bandwidth, low latency, and high reliability, has become the core technology for next-generation in-vehicle communication networks. This study focuses on bandwidth waste caused by guard bands and the limitations of Frame Pre-Emption in fully utilizing available bandwidth in In-Vehicle Ethernet. It aims to optimize TSN scheduling mechanisms by enhancing scheduling flexibility and bandwidth utilization, rather than modeling system-level vehicle functions. Based on the Time-Sensitive Networking (TSN) protocol, this paper proposes an innovative Adaptive Frame Segmentation (AFS) algorithm. The AFS algorithm enhances the performance of In-Vehicle Ethernet message transmission through flexible frame segmentation and efficient message scheduling. Experimental results indicate that the AFS algorithm achieves an average local bandwidth utilization of 94.16%, improving by 4.35%, 5.65%, and 30.48% over Frame Pre-Emption, Packet-Size Aware Scheduling (PAS), and Improved Qbv algorithms, respectively. The AFS algorithm demonstrates stability and efficiency in complex network traffic scenarios, reducing bandwidth waste and improving In-Vehicle Ethernet’s real-time performance and responsiveness. This study provides critical technical support for efficient communication in intelligent connected vehicles, further advancing the development and application of In-Vehicle Ethernet technology.

## 1. Introduction

The in-vehicle bus network system is the cornerstone of modern automotive electronic and electrical architecture, responsible for transmitting data and control signals among different Electronic Control Units (ECUs). With the rapid advancement of automotive technology, modern smart vehicles are equipped with various sensors, such as cameras, LiDAR, and radar, which generate large amounts of real-time data. To accommodate these increasing data demands, in-vehicle bus systems have undergone significant evolution. Traditional bus systems, such as the Local Interconnect Network (LIN), Controller Area Network (CAN), FlexRay, and Media-Oriented Systems Transport (MOST), have played a crucial role in earlier vehicle networks [1,2]. However, with the emergence of advanced functionalities like intelligent driving, in-car entertainment systems, and Advanced Driver Assistance Systems (ADAS), traditional bus systems have exhibited limitations, such as low transmission rates and insufficient real-time performance. These shortcomings make them unable to meet the high-bandwidth, low-latency communication demands of intelligent connected vehicles. As a next-generation bus technology, In-Vehicle Ethernet, leveraging its advantages of high bandwidth, low latency, and high reliability, has gradually replaced traditional bus systems and become the mainstream choice for in-vehicle communication [3,4,5]. By employing Time-Sensitive Networking (TSN) technology, In-Vehicle Ethernet enhances the real-time and deterministic characteristics of message transmission, achieving precise control over time-sensitive traffic [6]. Based on the IEEE 802 series of Ethernet technical standards, In-Vehicle Ethernet supports various topologies, such as point-to-point, star, and daisy-chain, providing unprecedented flexibility to in-vehicle networks and facilitating the expansion and integration of automotive electronic control systems [7].

TSN is a series of technical standards developed by the IEEE 802.1 Working Group. When combined with the IEEE 802.3 standard in In-Vehicle Ethernet, TSN enhances Ethernet’s time-sensitive capabilities to meet the stringent scheduling demands of various traffic types in-vehicle networks. However, while TSN ensures limited latency and jitter, it also faces the issue of suboptimal bandwidth utilization. Jitter refers to the variation in end-to-end transmission delay for periodic traffic, which can impact the predictability of time-sensitive communications. The Time-Aware Shaper (TAS) mechanism in TSN uses guard bands to prevent frame collisions. However, the use of guard bands can lead to bandwidth resource waste, especially when TAS and non-TAS windows switch frequently, resulting in increased usage of guard bands and exacerbating bandwidth wastage [8]. Extensive use of guard bands reduces network bandwidth utilization, increases delays for low-priority traffic, causes network congestion and message queue buildup, and impacts the flexibility of message scheduling, ultimately weakening the overall performance and stability of in-vehicle networks. Therefore, reducing bandwidth wastage caused by guard bands is a critical challenge for improving the practical performance of TSN in-vehicle networks.

The improved IEEE 802.1Qbv standard permits continuous frame transmission within guard bands until the remaining bandwidth in the non-TAS window can no longer accommodate a complete frame [9]. The IEEE 802.1Qbu and IEEE 802.3br standards introduce Frame Pre-Emption, allowing the interruption of ongoing low-priority frame transmissions to prioritize high-priority frames, thereby reducing the bandwidth occupied by guard bands [10]. J. Lee and S. Park proposed analytical methods to optimize guard band length, achieving bandwidth waste reduction by adjusting the length of guard bands [11]. Chuwen Zhang and Yi Wang integrated PAS with TSN to improve bandwidth utilization within guard bands [12].

Frame Pre-Emption effectively mitigates bandwidth waste caused by guard bands. However, current research lacks message scheduling methods tailored to Frame Pre-Emption, resulting in the underutilization of its advantages. Moreover, existing mainstream message scheduling algorithms treat frames of different lengths uniformly, failing to implement more refined management based on frame length differences. To address these issues, this study proposes an Adaptive Frame Segmentation (AFS) algorithm for efficient real-time data transmission in In-Vehicle Ethernet. Centered on the TAS and Frame Pre-Emption mechanisms of TSN, the AFS algorithm categorizes frames based on their lengths and dynamically optimizes frame segmentation strategies. This approach effectively resolves bandwidth resource wastage within guard bands, overcomes the constraints imposed by frame length on Frame Pre-Emption, and ensures minimal transmission delay.

The remainder of this paper is organized as follows: Section 2 introduces the key technologies of In-Vehicle Ethernet relevant to this study, including Time-Aware Shaping and Frame Pre-Emption. Section 3 provides a detailed description of the Adaptive Frame Segmentation (AFS) algorithm. Section 4 evaluates the performance of the AFS algorithm. Section 5 discusses the contributions and innovations of the AFS algorithm. Finally, Section 6 concludes the research and presents key findings.

## 2. Key Technologies of In-Vehicle Ethernet

Recent studies have extended the application of Time-Sensitive Networking (TSN) from the automotive sector to other critical domains, including aerospace and space launch systems. In this context, Fiori et al. proposed a simplified TSN solution specifically designed for space launch networks. Their research demonstrated the feasibility of applying TSN in highly constrained and real-time-critical aerospace systems, providing valuable insights into lightweight TSN deployment strategies. The application of TSN in the aerospace field further highlights its essential role in realizing lightweight and deterministic communication architectures in multiple domains [13]. This is also applicable to In-Vehicle Ethernet systems with stringent real-time requirements.

The current TSN standards primarily focus on three types of traffic: scheduled traffic, audio/video (A/V) traffic, and best-effort traffic. Scheduled traffic is characterized by its periodicity and predictable size, with stringent requirements for latency and jitter, making it suitable for vehicle control and critical tasks. A/V traffic is bursty, requires high bandwidth, and has strict constraints on jitter and packet loss rates. Best-effort traffic does not have stringent real-time requirements, but its scheduling may impact the transmission efficiency of higher-priority traffic [14,15], where frame transmission efficiency refers to the proportion of link bandwidth effectively occupied by valid payload data during a given time window. The TSN Task Group has introduced multiple related standards to manage scheduled traffic, audio/video (A/V) traffic, and best-effort traffic by allocating transmission time slots and priorities for different types of traffic. Each standard addresses specific requirements and provides corresponding technical support. For instance, the IEEE 802.1Qbv standard introduces the Time-Aware Shaper (TAS) to ensure isolated transmission for high-priority scheduled traffic. The IEEE 802.1Qbu and IEEE 802.3br standards collectively define Frame Pre-Emption, which enables flexible adjustments to the transmission sequence of frames based on their priorities. By combining TAS and Frame Pre-Emption, high-priority, time-sensitive traffic can be shielded from interference by lower-priority traffic, enabling efficient transmission of different traffic types in in-vehicle networks and achieving optimal resource allocation.

### 2.1. Time-Aware Shaper (TAS)

The fundamental requirement for TAS operation is clock synchronization across all nodes in the network, allowing time to be precisely divided into fixed-length windows ranging from microseconds to milliseconds. Each time window is periodically allocated to traffic of different priorities according to a predefined schedule, ensuring that only frames with specific priorities can be transmitted within a given time window. This guarantees low latency and isolated transmission for high-priority traffic.

**Figure 1:**
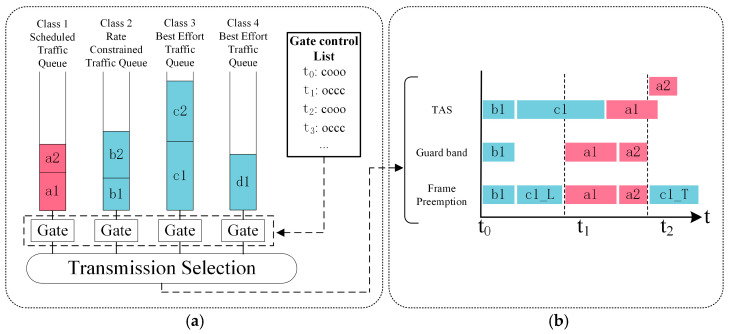
Example of TSN message transmission based on four queues: (**a**) TSN with four queues; (**b**) Message transmission example based on TAS, guard Band, and Frame Pre-Emption.

Figure 1 illustrates a TSN message transmission example based on four queues. In Figure 1a, traffic queues are classified by priority and traffic type, including the Class 1 scheduled traffic queue, the Class 2 rate-constrained traffic queue, and the Class 3 and Class 4 best-effort traffic queues. The transmission of each queue’s traffic is controlled by an independent gate. The Gate Control List (GCL), which forms the core control mechanism of TAS, comprises a series of time points and corresponding gate states, enabling precise control over the opening and closing of gates for each priority queue [16,17]. In Figure 1b, t1 and t2 denote the switching points between TAS and non-TAS windows. Frames in the buffer are transmitted in a First-In-First-Out (FIFO) order. At t1, the gate for the Class 1 scheduled traffic queue opens, while the gates for other queues close (occc). During the TAS window from t1 to t2, only high-priority scheduled traffic is allowed to transmit, monopolizing the bus resources, while low-priority traffic is completely isolated. However, if a low-priority frame (c1) in the buffer is still being transmitted at t1, the high-priority frame (a1) cannot begin transmission immediately, even though the Class 1 queue’s gate is open. This delay in transmitting frame a1 further prevents frame a2 from being transmitted within the allocated window, forcing it to be deferred to the next TAS window. As delays accumulate for the front-end high-priority frames, the transmission delay for back-end high-priority frames progressively increases, leading to greater network uncertainty and increased network jitter. If the low-priority frame completes transmission before the start of the TAS window, these high-priority frame delays can be avoided.

The IEEE 802.1Qbv standard introduces a guard band at the beginning of each TAS window, with a length equal to the maximum Ethernet frame size. As shown in Figure 1b, labeled as “Guard band”, the guard band prohibits the initiation of any new frame transmission but allows ongoing frame transmissions to complete [18]. The guard band ensures the timely transmission of high-priority frames within the TAS window but results in significant bandwidth waste. The IEEE 802.1Qbu standard introduces Frame Pre-Emption to enhance bandwidth utilization further, as shown in Figure 1b, labeled as “Frame Preemption”. Frame Pre-Emption partially mitigates the bandwidth waste caused by the guard band; however, its implementation is still constrained by the length of low-priority frames and the remaining bandwidth in the non-TAS window.

### 2.2. Frame Pre-Emption

Traditional In-Vehicle Ethernet employs a FIFO mechanism, where frames are transmitted in the order they arrive. If a low-priority frame is being transmitted and a high-priority frame arrives in the queue buffer, the high-priority frame must wait for the low-priority frame to finish transmission before it can begin. Under the FIFO mechanism, high-priority and time-sensitive data may experience significant delays and jitter. Frame Pre-Emption allows high-priority frames to interrupt the transmission of low-priority frames. When high-priority data arrives, the ongoing transmission of a low-priority frame is interrupted and divided into a transmitted segment and an untransmitted segment. Once the high-priority frame transmission is completed, the untransmitted segment of the low-priority frame resumes, ensuring data integrity and avoiding data loss or retransmission. Frame Pre-Emption ensures the real-time transmission of high-priority traffic and allows low-priority traffic to fill idle bandwidth without compromising the timely delivery of critical traffic, thereby preventing bandwidth waste [19,20].

In-Vehicle Ethernet Frame Pre-Emption is defined by two standards: IEEE 802.3br and IEEE 802.1Qbu. The IEEE 802.3br standard primarily specifies the fragmentation and reassembly mechanism for low-priority frames to ensure uninterrupted transmission of fragmented frames after pre-emption. The IEEE 802.1Qbu standard defines the conditions for triggering pre-emption and provides overall behavior control for priority scheduling. As this study focuses on the impact of Frame Pre-Emption on bandwidth utilization, the analysis centers on the IEEE 802.3br standard [21].

The IEEE 802.3br standard classifies network traffic into Express Traffic and Pre-Emptable Traffic. Express Traffic has higher priority and cannot be interrupted during transmission. Pre-Emptable Traffic, with lower priority, can be interrupted by Express Traffic during transmission. Notably, Pre-Emptable Traffic cannot be interrupted by other Pre-Emptable Traffic, regardless of its priority, ensuring the stability of Frame Pre-Emption and simplifying traffic management [22]. To maintain compatibility with existing In-Vehicle Ethernet devices, the IEEE 802.3br standard retains the basic structure of MAC frames defined in the IEEE 802.3 standard, ensuring frames can be appropriately transmitted at the physical layer (PHY). Consequently, during Frame Pre-Emption, each segment of a Pre-Emptable MAC Frame must have a valid In-Vehicle Ethernet frame format that satisfies the PHY’s frame transmission requirements.

**Figure 2:**
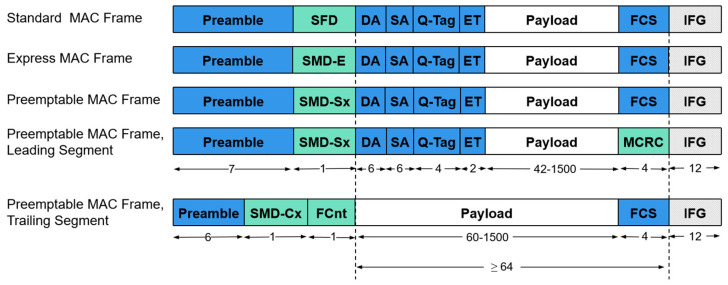
Standard IEEE 802.3 MAC frame format and four IEEE 802.3br frame formats (all values are in bytes).

The IEEE 802.3br standard further optimizes and extends the MAC frame format. As shown in Figure 2, the IEEE 802.3br standard defines multiple MAC frame formats. All MAC frames begin with a preamble and end with either a Frame Check Sequence (FCS) or a Modified Frame Check Sequence (MCRC), followed by an Interframe Gap (IFG). A standard IEEE 802.3 MAC frame consists of nine fields. The preamble is used to synchronize devices on the transmitting and receiving networks, helping the receiver lock onto the transmission clock to prepare for correct frame reception. The Start Frame Delimiter (SFD) marks the beginning of the frame. The Destination Address (DA) and Source Address (SA) indicate the recipient and sender of the data, respectively. The IEEE 802.1Q tag (Q-Tag) supports VLAN and priority control. The EtherType (ET) field specifies the protocol type of the frame. The payload field carries application layer data. The Frame Check Sequence (FCS) detects transmission errors in the frame. The Interframe Gap (IFG) provides sufficient time for network devices to prepare for the transmission and reception of the next frame. To support Frame Pre-Emption, the IEEE 802.3br standard introduces additional delimiters and counters. Specifically, the Start MFrame Delimiter-Express (SMD-E) marks the beginning of Express Traffic frames. The Start Fragment Delimiter (SMD-Sx) indicates the starting segment of Pre-Emptable Traffic frames. The Continuation Fragment Delimiter (SMD-Cx) identifies consecutive segments of low-priority frames. The Fragment Counter (FCnt) assists the receiver in correctly sequencing and reassembling frame fragments [23].

All Pre-Emptable MAC Frames must meet the minimum length requirements for Ethernet frames, specifically that the length of a fragmented frame segment must exceed 84 bytes. As this study focuses on utilizing Frame Pre-Emption to fill the remaining bandwidth in the guard band, Pre-Emptable Frames are divided into no more than two segments. Moreover, Frame Pre-Emption does not permit padding of fragmented frame segments. This introduces two key constraints for Frame Pre-Emption: (a) The transmitted segment of a Pre-Emptable Frame must be larger than 84 bytes. (b) The total length of a Pre-Emptable Frame must not be less than 144 bytes. These constraints, a and b, reduce the flexibility of low-priority frames in filling unused bandwidth within the guard band, leading to a decrease in bandwidth utilization and an increase in transmission delay. Mitigating the impact of constraints a and b on Frame Pre-Emption can enhance bandwidth utilization and optimize the allocation efficiency of bandwidth resources before and after the TAS window.

## 3. Adaptive Frame Segmentation Algorithm

To further optimize the message scheduling performance of In-Vehicle Ethernet, this study proposes the Adaptive Frame Segmentation (AFS) algorithm, aiming to overcome the bottlenecks in bandwidth utilization and Frame Pre-Emption efficiency present in existing mechanisms. The AFS algorithm employs dynamic frame segmentation and refined management to enable the communication network to adaptively adjust transmission strategies under various traffic scenarios, thereby improving overall network efficiency.

### 3.1. Principles of the AFS Algorithm

In In-Vehicle Ethernet systems, the remaining bandwidth r in the non-TAS traffic window is dynamically variable. The AFS algorithm is applied within the critical range 84 byte < r < 1542 byte. By effectively utilizing the bandwidth resources within this critical range, the algorithm allows previously wasted fragment bandwidth caused by window transitions or frame length constraints to carry more valid payload, thereby improving frame transmission efficiency. The lower limit of 84 bytes is chosen because bandwidth less than 84 bytes cannot support the transmission of a complete frame. The upper limit of 1542 bytes corresponds to the maximum transmission unit (MTU) defined by In-Vehicle Ethernet standards. The optimization performance of the AFS algorithm is most significant within this range.

**Figure 3:**
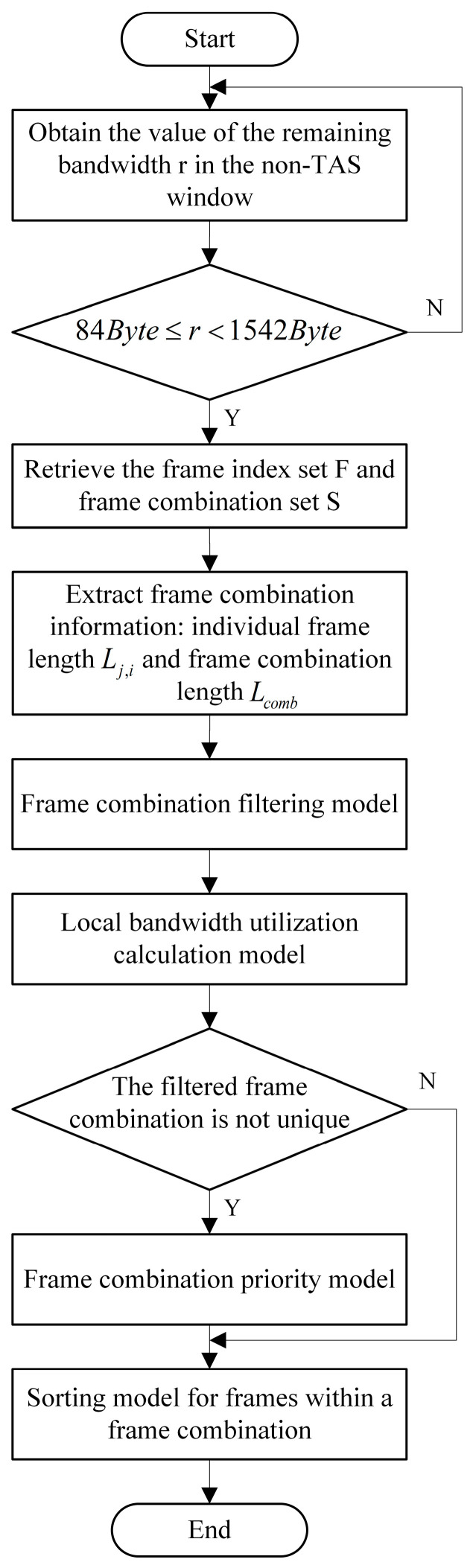
Flowchart of the AFS algorithm.

Figure 3 illustrates the flowchart of the AFS algorithm. The algorithm comprises four models: the Frame Combination Filtering Model, the Local Bandwidth Utilization Calculation Model, the Frame Combination Priority Model, and the Sorting Model for Frames Within a Frame Combination. The process begins by acquiring the value of the remaining bandwidth r in the current non-TAS window and determining whether r falls within the range of 84 to 1542 bytes. If not, the algorithm rechecks the bandwidth. If the condition is met, the algorithm retrieves the frame index set F and frame combination set S. It extracts information from the sets, including the length $L_{j,i}$ of each frame and the total length $L_{comb}$ of frame combinations. Using the Frame Combination Filtering Model, the algorithm screens frame combinations based on specific constraints. For the selected frame combinations, the Local Bandwidth Utilization Calculation Model is used to determine the local bandwidth utilization, and the frame combination with the highest utilization is selected. If multiple frame combinations exhibit the same bandwidth utilization, the Priority Model is employed to further refine the selection, ensuring that the frame combination with the highest priority is chosen for transmission under equivalent conditions. Finally, the frames within the selected frame combination are sorted before transmission.

The AFS algorithm selects several frames from the buffer of non-TAS traffic queues, defined as a frame combination s. Let m denote the total number of non-TAS traffic queues, $f_{j,i}$ represent the i-th frame in queue j, and $n_{j}$ represent the total number of queued frames in queue j. The frame index set is defined as shown in Equation (1).

(1) $$F=\{(j,i)|1\leq j\leq m,1\leq i\leq n_{j}\}.$$

The set F contains the index pairs of all frames in the buffer. A binary variable $x_{j,i}$ is used to indicate whether a frame $f_{j,i}$ is selected into the frame combination s. The length of the frame combination $L_{comb}$ is defined as shown in Equation (2).

(2) $$L_{comb}=\sum_{(j,i)\in F}x_{j,i}L_{j,i}.$$

where $L_{j,i}$ represents the length of frame $f_{j,i}$. Further, a frame combination set S is defined, which includes all possible frame combinations. The AFS algorithm optimizes frame selection by determining the value of $x_{j,i}$ corresponding to each frame $f_{j,i}$, thereby improving bandwidth utilization. Additionally, the longest frame $f_{k,l}$ within a frame combination has a significant impact on the transmission efficiency of the combination. Therefore, the AFS algorithm incorporates specific optimizations for the selection and scheduling of $f_{k,l}$. The symbols and their corresponding functions used in this paper are summarized in Table 1.

### 3.2. Frame Combination Filtering Model

**Figure 4:**
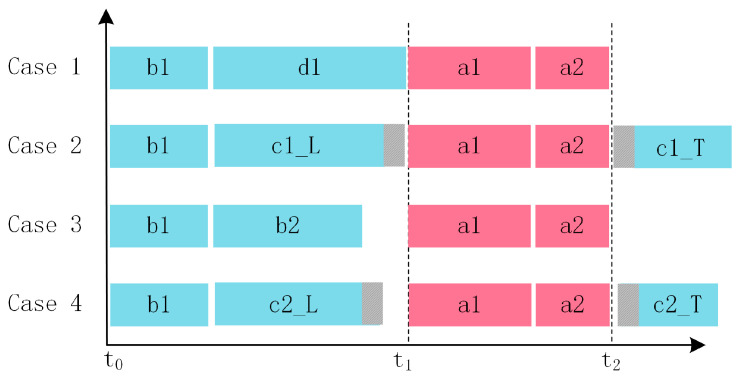
Frame transmission results based on four different frame combinations (priority order: a > b > c > d).

The AFS algorithm employs a strategy of classifying and managing frame combinations. To enable the AFS algorithm to flexibly adapt to various traffic scenarios under limited bandwidth resources, frame combinations are categorized into four cases (Case 1–Case 4) based on the remaining bandwidth r in the non-TAS traffic window and the total length of the frame combination, as illustrated in Figure 4. In Figure 4, frames a, b, c, and d represent different candidate data frames with descending transmission priority, where frame a has the highest priority and frame d the lowest.

In scenarios where frame segmentation is not involved, the bandwidth utilization is significantly higher when the total length of the frame combination is no less than r−16 Byte. Therefore, frame combinations that do not involve segmentation are further divided into Case 1 and Case 3. To refine the optimization of frame transmission strategies in In-Vehicle Ethernet, this study defines the specific constraints for Cases 1 to 4 in descending order of bandwidth utilization, providing a more granular basis for optimization. It can be observed that since the AFS algorithm is built upon the Frame Pre-Emption mechanism, it naturally inherits its key advantage, which is that non-TAS low-priority frames scheduled by AFS do not interfere with the transmission of high-priority TAS frames under any of the four defined scheduling cases.

Case 1: Within the time window from t0 to t1, the frame combination consists of b1 and d1, where the last frame, d1, is completed precisely before t1 without requiring segmentation. The length of the frame combination in Case 1 satisfies Equation (3), ensuring that the frame combination in Case 1 can fully utilize the remaining bandwidth in the non-TAS flow window without segmenting any frames.

(3) $$r-16\;Byte\leq L_{comb}\leq r.$$

Case 2: Within the time window from t0 to t1, the frame combination includes b1 and c1. Since the total length of the frame combination exceeds the remaining bandwidth, c1 cannot be fully transmitted before t1 and thus requires segmentation. After segmentation, the leading segment c1_L of c1 is transmitted within the non-TAS window before t1, while the trailing segment c1_T is deferred to the non-TAS window after t2. The length of the frame combination in Case 2 satisfies Equations (4) and (5), ensuring that both segments of the segmented frame are at least 84 bytes long.

(4) $$r+60\;Byte\leq L_{comb}.$$

(5) $$L_{rest}\leq r-84\;Byte.$$

Case 3: Within the time window from t0 to t1, the frame combination includes b1 and b2. Frame b2 completes transmission before t1, but the remaining bandwidth is not fully utilized. The frame combination length for Case 3 is described by Equation (6). The frame combination in Case 3 does not involve complex frame segmentation operations and fails to fully utilize the remaining bandwidth in the non-TAS window, resulting in a lower bandwidth utilization compared to Cases 1 and 2.

(6) $$84\;Byte\leq L_{comb}\leq r-16\;Byte.$$

Case 4: Within the time window from t0 to t1, the frame combination includes b1 and c2. Frame c2 cannot be fully transmitted within the non-TAS window and must be segmented. If the Frame Pre-Emption mechanism is used to segment c2 at t1, the length of the trailing segment c2_T will fail to meet the minimum requirement of 84 bytes. To ensure that the segmented frame parts satisfy the minimum length requirement of 84 bytes, the AFS algorithm adopts a more flexible frame segmentation strategy. Based on real-time network conditions, the AFS algorithm examines the remaining length of the low-priority frame before the window switch point t1. If the remaining length does not meet the requirement, the transmission of the low-priority frame is pre-emptively interrupted before t1, ensuring that the trailing segment of the segmented frame has a length equal to 84 bytes. This approach effectively addresses the limitation of standard Frame Pre-Emption mechanisms at t1, where segmentation would otherwise be infeasible, thereby improving bandwidth utilization. The precise conditions for segmentation and transmission are defined by Equations (7) and (8).

(7) $$r<L_{comb}<r+60\;Byte.$$

(8) $$L_{k,l}\geq 144\;Byte.$$

By integrating the constraints of the four cases outlined above, frame combinations can be classified and filtered, resulting in the AFS algorithm’s frame combination selection model, as described by Equations (9)–(12). Specifically, Equations (9), (11), and (12) are respectively used to select the frame combination with the longest $L_{comb}$ from Cases 1, 3, and 4. Equation (10) is used to filter all frame combinations that meet the conditions of Case 2.

(9) $$max\{\sum_{(j,i)\in F}x_{j,i}L_{j,i}|r-16\leq \sum_{(j,i)\in F}{x_{j,i}L}_{j,i}\leq r,x_{j,i}\in \{0,1\}\}.$$

(10) $$\{\sum_{\left(j,i\right)\in F}x_{j,i}L_{j,i}|\sum_{\left(j,i\right)\in F,\left(j,i\right)\neq \left(k,l\right)}{x_{j,i}L}_{j,i}\leq r-84,\sum_{\left(j,i\right)\in F}{x_{j,i}L}_{j,i}\geq r+60,x_{j,i}\in \left\{0,1\right\}\}.$$

(11) $$max\{\sum_{(j,i)\in F}x_{j,i}L_{j,i}|84\leq \sum_{(j,i)\in F}{x_{j,i}L}_{j,i}\leq r-16,x_{j,i}\in \{0,1\}\}.$$

(12) $$max\{\sum_{(j,i)\in F}x_{j,i}L_{j,i}|r<\sum_{(j,i)\in F}{x_{j,i}L}_{j,i}<r+60,\sum_{\left(j,i)\in F,(j,i)=(k,l\right)}{x_{j,i}L}_{j,i}\geq 144,x_{j,i}\in \{0,1\}\}.$$

### 3.3. Local Bandwidth Utilization Calculation Model

The local bandwidth utilization represents the ratio of the actual transmitted data to the available bandwidth within a specific bandwidth range. It reflects the efficiency of bandwidth usage during frame transmission. To enhance the bandwidth utilization within the guard band, this study proposes a local bandwidth utilization calculation model aimed at selecting the frame combination with the highest local bandwidth utilization.

**Figure 5:**
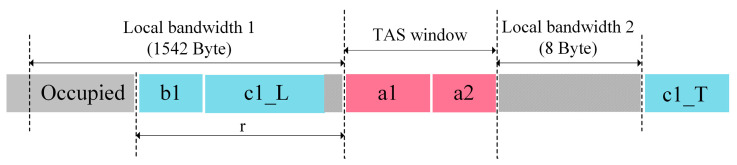
Local bandwidth structure diagram (Case 2).

Figure 5 illustrates the structure of the local bandwidth for Case 2, which consists of three parts: Local Bandwidth 1, the TAS window, and Local Bandwidth 2. Among them, Local Bandwidth 1 has a capacity of 1542 bytes. The AFS algorithm optimizes the transmission strategy within Local Bandwidth 1 to improve the efficiency of bandwidth resource utilization. Local Bandwidth 2, with a capacity of 8 bytes, is used during Frame Pre-Emption to transmit the header of the trailing segment (c1_T) of a pre-empted frame.

Equation (13) represents the local bandwidth utilization $\eta_{local}$ within the Local Bandwidth 1 and Local Bandwidth 2 regions. Here, $D_{actual}$ denotes the actual transmitted data, while $B_{local1}$ and $B_{local2}$ represent the bandwidth capacities of the Local Bandwidth 1 and Local Bandwidth 2 regions, respectively.

(13) $$\eta_{local}=\frac{D_{actual}}{B_{local1}+B_{local2}}\times 100\%.$$

In the local bandwidth, the utilized bandwidth is $\left(1542-r\right)$ bytes. For frame combinations in Case 1 and Case 3, the TAS window’s subsequent 8-byte bandwidth (Local Bandwidth 2) is not consumed. Consequently, this 8-byte segment is included in the actual transmitted data $D_{actual}$. The local bandwidth utilization for frame combinations in Case 1 and Case 3 is described by Equation (14).

(14) $$\eta_{local(1,3)}=\frac{\left(1542-r\right)+L_{comb}+8}{1542+8}.$$

Frame combinations in Case 2 and Case 4 involve Frame Pre-Emption. During the segmentation of pre-emptable frames, the frame’s leading segment is appended with a 16-byte trailer, and its trailing segment is prepended with an 8-byte header. Thus, an additional 24 bytes of data are added to the original frame. Since these 24 bytes are not part of the original frame’s payload, they are excluded from the actual transmitted data $D_{actual}$ when calculating the local bandwidth utilization $\eta_{local}$. Frame combinations in Case 2 and Case 4 adopt different local bandwidth utilization formulas due to their varying characteristics. The utilized bandwidth in the local bandwidth remains (1542−r) byte.

Equation (15) provides the local bandwidth utilization formula for frame combinations in Case 2. Notably, the local bandwidth utilization for Case 2 remains constant.

(15) $$\eta_{local(2)}=\frac{\left(1542+8\right)-(16+8)}{1542+8}=\frac{1542-16}{1542+8}.$$

Equation (16) describes the local bandwidth utilization formula for frame combinations in Case 4. The AFS algorithm dynamically adjusts the frame segmentation position, ensuring that the length of the trailing segment of the pre-emptable frame is fixed at 84 bytes (8-byte header + 76-byte payload), while the length of the leading segment becomes $\left(L_{comb}-76\right)$ byte.

(16) $$\eta_{local(4)}=\frac{\left(1542-r)+(L_{comb}-76\right)}{1542+8}.$$

By combining Equations (14)–(16), the local bandwidth utilization model is derived in Equation (17), enabling the selection of the frame combination with the highest local bandwidth utilization.

(17) $$max\{\eta_{local}|\eta_{local\left(1,3\right)},\eta_{local\left(2\right)},\eta_{local(4)}\}.$$

### 3.4. Frame Combination Priority Model and Sorting Model for Frames Within a Frame Combination

The frame combination priority ($p_{comb}$) refers to the average priority of all frames within a given frame combination, providing a comprehensive evaluation of the overall priority level of the combination, as shown in Equation (18). Here, $p_{j,i}$ denotes the priority of the i-th frame in queue j. The frame combination priority plays a crucial role in determining the transmission order during scheduling, ensuring that frame combinations with higher priorities are transmitted first.

(18) $$p_{comb}=\frac{\sum_{(j,i)\in F}x_{j,i}p_{j,i}}{\sum_{(j,i)\in F}x_{j,i}}.$$

To select the frame combination with the highest priority from those with the same bandwidth utilization, this study proposes a Frame Combination Priority Model, as expressed in Equation (19).

(19) $$min\{p_{comb}|p_{comb}=\frac{\sum_{(j,i)\in F}x_{j,i}p_{j,i}}{\sum_{(j,i)\in F}x_{j,i}}\}.$$

**Figure 6:**
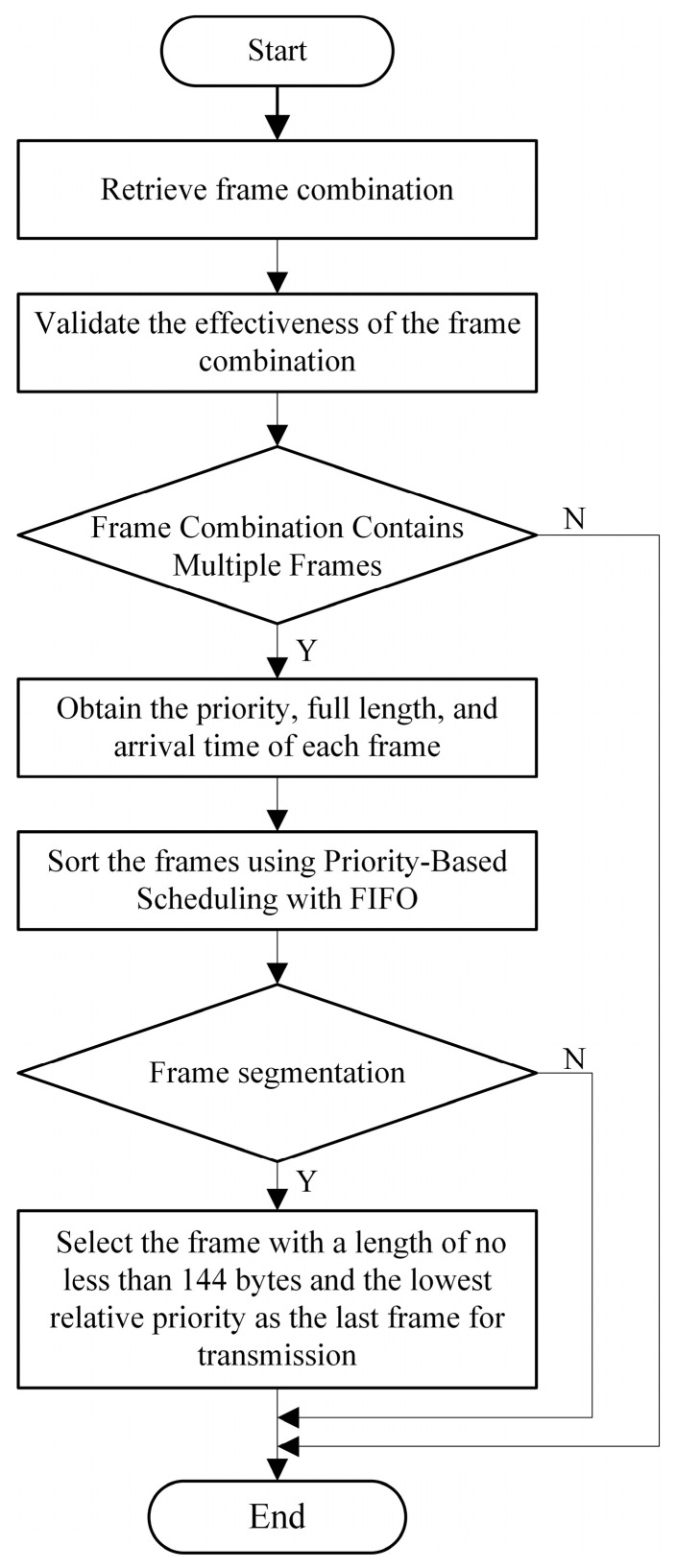
Flowchart of sorting model for Frames Within a Frame Combination.

After selecting the frame combination with the highest priority, the frames within the combination are sorted. The sorting process for Frames Within a Frame Combination is illustrated in Figure 6.

First, retrieve the frame combination that passed the screening process and verify its validity. If the frame combination contains only one frame, it is transmitted directly. If the frame combination contains multiple frames, further details such as each frame’s priority, complete length, and arrival time are obtained. Reorder the Frames Within a Frame Combination using priority-based FIFO scheduling. Frames with higher priority are transmitted first. If multiple frames share the same priority, they are scheduled based on their arrival time in the buffer, following the FIFO principle. When frame segmentation is not involved, the frames are transmitted according to the reordered sequence. If the frame combination involves frame segmentation, the frame with a length of at least 144 bytes and the lowest relative priority within the combination is selected as the last frame to be transmitted, ensuring successful frame segmentation.

## 4. Simulation Experiments

**Figure 7:**
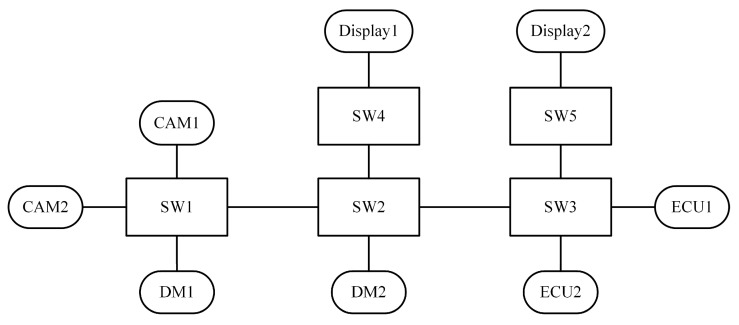
Network topology architecture of In-Vehicle Ethernet.

CANoe (Vector Informatik GmbH, Stuttgart, Germany) is a professional simulation and analysis platform widely adopted in the development and validation of in-vehicle communication systems. Its Ethernet extension module, CANoe-Ethernet, provides strong support for Time-Sensitive Networking (TSN) simulation and is extensively used by OEMs and suppliers to evaluate TSN-based architectures [24,25]. In this study, CANoe-Ethernet was used to conduct experimental simulations to ensure the credibility of the results. To accurately reflect the communication requirements and message characteristics of real vehicles, this study simulates a realistic in-vehicle communication scenario based on Ethernet traffic profiles provided by Renault. In addition, considering the data transmission characteristics of automotive sensors, a network topology was designed as shown in Figure 7. The structure was inspired by a prototype architecture from Renault to ensure the realism of the simulation environment.

The network topology consists of five TSN-capable switches (SW1–SW5) and ten end systems, including two cameras (CAM1 and CAM2), two displays (Display1 and Display2), two Electronic Control Units (ECU1 and ECU2), and two domain masters (DM1 and DM2). These nodes are interconnected via 100 Mbps full-duplex links, forming a typical multi-domain In-Vehicle Ethernet architecture. The traffic scenario reflects a representative in-vehicle communication environment, which includes multiple periodic data streams initiated by sensors, control command exchanges between domain controllers and ECUs, and bidirectional data flows between cameras and displays. This configuration enables a more accurate evaluation of the proposed algorithm under realistic automotive network conditions.

The simulated message set, summarized in Table 2, is based on a statistical abstraction of real in-vehicle traffic profiles provided by Renault and captures a diverse range of communication requirements.

In the experimental evaluation, we selected three representative scheduling algorithms, Frame Pre-Emption, Improved Qbv, and Packet-Size Aware Scheduling (PAS), as benchmark baselines. These three methods reflect the mainstream optimization strategies in the TSN scheduling domain, focusing on improving bandwidth utilization and scheduling flexibility.

Frame Pre-Emption allows high-priority frames to pre-empt low-priority ones, which effectively mitigates the bandwidth waste caused by TAS window transitions. Improved Qbv, as an enhancement to the existing Qbv mechanism, was specifically designed to address potential bandwidth underutilization during the transition phases between scheduling windows, which is highly aligned with the objective of this study. This also highlights the necessity of improving the utilization efficiency of fragmented bandwidth by low-priority messages. PAS is a classical scheduling algorithm inspired by the Precedence-Constrained Knapsack Problem (PCKP). It optimizes frame group selection based on frame length and priority in order to maximize bandwidth utilization, especially in the guard bands introduced by Time-Aware Shaping (TAS).

These three algorithms are representative in both theoretical research and engineering practice and are therefore chosen as the benchmark comparison group in this study.

Table 3 presents a comparative analysis of local bandwidth utilization between the proposed AFS algorithm and the Frame Pre-Emption, PAS, and Improved Qbv algorithms. The results indicate that the AFS algorithm consistently achieves higher local bandwidth utilization than the other three algorithms.

**Figure 8:**
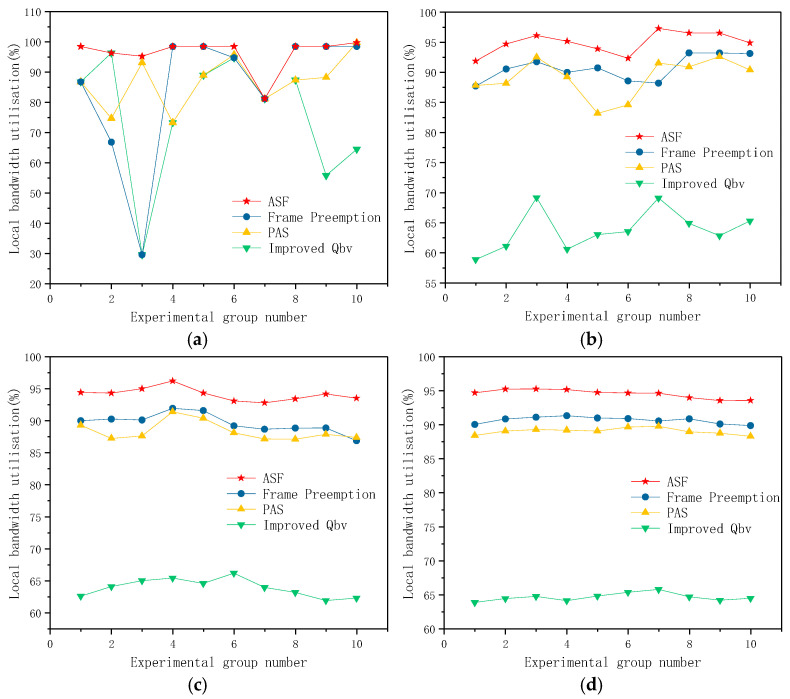
Local bandwidth utilization results based on the message configuration in Table 2: (**a**) local bandwidth utilization corresponding to the results in Table 3; (**b**) local bandwidth utilization averaged over every 50 groups; (**c**) local bandwidth utilization averaged over every 200 groups; (**d**) local bandwidth utilization averaged over every 400 groups.

Figure 8 compares the local bandwidth utilization of four algorithms: AFS, Frame Pre-Emption, PAS, and Improved Qbv. Specifically, Figure 8a illustrates the local bandwidth utilization results for 10 experimental data sets. The AFS algorithm demonstrates high performance stability, which in this context refers to the low variance in bandwidth utilization observed across multiple experimental scenarios. In contrast, the local bandwidth utilization of the other three algorithms exhibits significant jitter, making it challenging to evaluate their overall performance reliably.

To further compare the overall performance of each algorithm, the experimental data was averaged over different scales. Figure 8b–d present the averaged results for every 50 sets, 200 sets, and 400 sets of experimental data, respectively. As the amount of experimental data increases, the bandwidth utilization stabilizes when averaged over 400 data sets.

This study conducted simulation experiments on 1000 sets of experimental data. By averaging the results from 1000 sets of experimental data, the average local bandwidth utilization of the four algorithms, AFS, Frame Pre-Emption, PAS, and Improved Qbv, is presented in Table 4. The averages of experimental data at different scales in Figure 8 gradually stabilize and converge to the values presented in Table 4. Therefore, the conclusions in Table 4 are reliable and representative. The AFS algorithm achieves an average local bandwidth utilization of 94.16%, which is 4.35%, 5.65%, and 30.48% higher than those of the Frame Pre-Emption, PAS, and Improved Qbv algorithms, respectively. This result clearly reflects the efficiency of the algorithm, as it demonstrates a stronger ability to utilize available bandwidth resources under the same network conditions.

## 5. Discussion

Table 4 has clearly demonstrated the advantages of the AFS algorithm in terms of bandwidth utilization. Although the AFS algorithm primarily focuses on optimizing bandwidth utilization, we fully recognize that delay performance is a critical metric in TSN-based in-vehicle networks. AFS shares conceptual similarities with the PAS algorithm in terms of selecting frame groups based on frame length and priority. As a result of this similarity in selection logic, AFS does not introduce additional scheduling latency.

On top of this foundation, AFS further incorporates a case-based classification mechanism (Case 1–Case 4) that aligns with the Time-Aware Shaper (TAS) time slot structure and Frame Pre-Emption thresholds defined in TSN. This enables dynamic scheduling decisions that maximize bandwidth efficiency while reducing the risk of blocking high-priority frames during TAS window transitions. Therefore, although minimizing delay is not the primary objective of AFS, its overall strategy inherently helps maintain delay stability for time-sensitive traffic.

Compared with PAS, AFS not only achieves superior bandwidth efficiency but also demonstrates stronger compatibility with TSN’s deterministic scheduling mechanisms, making it more suitable for deployment in practical In-Vehicle Ethernet systems.

In real In-Vehicle Ethernet scenarios, fragmented bandwidth often arises within non-TAS windows due to frequent scheduling window transitions. Although this bandwidth cannot be used to transmit high-priority, time-sensitive flows, if left underutilized, its long-term accumulation leads to resource wastage, reduced scheduling flexibility, and congestion in low-priority traffic. Moreover, in network environments such as In-Vehicle Ethernet and TSN, which have high real-time requirements and limited resources, even a slight improvement in bandwidth utilization can have a long-term impact on system throughput, stability, and scheduling flexibility, especially when handling large-scale sensor data, image streams, and other real-time traffic. The Improved Qbv mechanism proposed by the IEEE 802.1 working group addresses these issues by introducing more complex logic to enhance the utilization of fragmented bandwidth. The AFS algorithm increases the bandwidth utilization of the non-TAS window without affecting the scheduling of high-priority messages, aligning with this objective.

Furthermore, while Frame Pre-Emption is widely adopted, as mentioned earlier, there are significant limitations in its practical application. The AFS algorithm mitigates these limitations by employing a classification scheduling strategy based on remaining bandwidth and frame combination length, thus improving the flexibility and adaptability of Frame Pre-Emption in non-TAS windows. Therefore, the contribution of AFS is not only reflected in the performance improvements demonstrated by the experimental results but also in its ‘adaptive frame segmentation’ scheduling concept. This research provides a new approach to enhancing scheduling flexibility within TSN.

In summary, the novelty of the proposed AFS algorithm lies in its integrated design that combines adaptive frame combination, dynamic segmentation, and in-frame sorting under TAS-pre-emptive coordination. This forms a scheduling enhancement module that is fully compatible with TSN and effectively addresses the bandwidth waste caused by TAS window transitions. Such improvements are essential for supporting high-throughput and time-sensitive communication in modern intelligent vehicle systems.

Although the AFS algorithm demonstrates excellent performance in bandwidth utilization, it still has several limitations. First, its performance depends on the remaining bandwidth falling within a specific range, between 84 and 1542 bytes. Outside this range, the optimization effect may be limited. Second, to reduce computational complexity, the algorithm does not consider frame deadlines. This limitation, however, can be mitigated by integrating AFS with a regulating scheduler. Finally, this study has not yet been validated on real In-Vehicle Ethernet hardware, and future work will involve platform-based testing to evaluate engineering feasibility and real-time performance.

## 6. Conclusions

This paper proposes an Adaptive Frame Segmentation (AFS) algorithm for In-Vehicle Ethernet. As an independent scheduling logic module within the TSN-based In-Vehicle Ethernet framework, AFS does not replace the standardized Time-Aware Shaper or Frame Pre-Emption mechanisms, but rather complements them by enhancing scheduling flexibility under TAS-pre-emptive coordination. To improve bandwidth utilization during TAS window transitions, AFS classifies and manages frames in the non-TAS window and selects frame combinations based on the remaining bandwidth and frame length. A flexible frame segmentation strategy is employed to reduce bandwidth waste. In addition, the Sorting Model for Frames Within a Frame Combination is used to adjust the transmission order, ensuring efficient and effective execution of frame segmentation. Experimental results show that the AFS algorithm achieves an average local bandwidth utilization of 94.16%, exhibiting stable and efficient performance. Moreover, the AFS design is fully compatible with TSN architecture and can serve as an embedded enhancement to extend the applicability and flexibility of the Frame Pre-Emption mechanism in real-time systems.

## Figures and Tables

**Table 1:** Symbols and their functional descriptions.

Symbol	Description
m	Number of queues in non-TAS traffic
$n_{j}$	Number of frames queued in queue j (j = 1, 2, …, m)
$f_{j,i}$	$\mathrm{The}\;\text{i-th}\;\mathrm{frame}\;\mathrm{in}\;\mathrm{queue}\;j\;(i=1,\;\ldots ,\;n_{j}$)
$L_{j,i}$	Length of the i-th frame in queue j
r	Remaining bandwidth in the non-TAS traffic window
F	Frame index set containing all frame indices (j,i) in the buffer
s	Frame combination
S	Set of frame combinations containing all possible combinations
$L_{comb}$	Length of the frame combination
$x_{j,i}$	$\mathrm{Binary}\;\mathrm{variable}\;\mathrm{indicating}\;\mathrm{whether}\;\mathrm{frame}\;f_{j,i}$ is included in the combination
$f_{k,l}$	Longest frame in the frame combination, (k,l)∈(j,i)
$L_{rest}$	$\mathrm{Total}\;\mathrm{length}\;\mathrm{of}\;\mathrm{other}\;\mathrm{frames}\;\mathrm{in}\;\mathrm{the}\;\mathrm{combination},\;\mathrm{excluding}\;f_{j,i}$
$p_{j,i}$	Priority of the i-th frame in queue j
x	Number of frames in the frame combination
$p_{comb}$	Priority of the frame combination

**Table 2:** Message set configuration for In-Vehicle Ethernet simulation.

Traffic Type	Number of Flows	Frame Size (bytes)	Period (ms)	Priority	Deadline Constraint
Scheduled Traffic	10	128–512	1–5	5–7	Hard
AVB Traffic	16	256–1024	10–20	3–4	Soft
Best-Effort Traffic	42	64–800	Aperiodic/Mixed	0–2	None/Soft

**Table 3:** Local bandwidth utilization for 10 sets of data.

	AFS (%)	Frame Pre-Emption (%)	PAS (%)	Improved Qbv (%)	AFS Case
1	98.45	86.77	86.77	86.77	2
2	96.32	66.83	74.70	96.32	3
3	95.22	29.61	93.09	29.61	4
4	98.45	98.45	73.29	73.29	2
5	98.45	98.45	88.96	88.96	2
6	98.45	94.70	95.74	94.70	2
7	81.16	81.16	81.16	81.16	3
8	98.45	98.45	87.35	87.35	2
9	98.45	98.45	88.25	55.80	2
10	99.74	98.45	99.74	64.51	1

**Table 4:** Average local bandwidth utilization.

AFS (%)	Frame Pre-Emption (%)	PAS (%)	Improved Qbv (%)
94.16%	89.81%	88.51%	63.68%

## Data Availability

The data that support the findings of this study are not publicly available due to privacy concerns. Requests for access to the data should be directed to the corresponding author.

## Abbreviations

The following abbreviations are used in this manuscript:

AFS	Adaptive Frame Segmentation
PAS	Packet-Size Aware Scheduling
TSN	Time-Sensitive Networking
TAS	Time-Aware Shaper
ECU	Electronic Control Unit
LIN	Local Interconnect Network
CAN	Controller Area Network
MOST	Media-Oriented Systems Transport
ADAS	Advanced Driver Assistance Systems
AVB	Audio Video Bridging
GCL	Gate Control List
FCS	Frame Check Sequence
IFG	Interframe Gap
SFD	Start Frame Delimiter
DA	Destination Address
SA	Source Address
Qbv	IEEE 802.1Qbv Standard
Qbu	IEEE 802.1Qbu Standard
SW	Switch
CAM	Camera
DM	Domain Master
PCKP	Precedence-Constrained Knapsack Problem
